# Novel Cancer Immunotherapies and Antitumor Immunity

**DOI:** 10.1155/2019/3742061

**Published:** 2019-07-22

**Authors:** Lei Zhao, Jianjun Zhang, Liang Xu, Hussein A. Abbas

**Affiliations:** ^1^PLA General Hospital, Beijing, China; ^2^The University of Texas, MD Anderson Cancer Center, Houston, USA; ^3^Beijing Institute of Pharmacology and Toxicology, Beijing, China

Recent success of immune-checkpoint blockade therapy in clinic has revealed that blockade of tumor-associated immunosuppression and initiation of tumor-specific immunity could be critical for the development of novel immunotherapies against cancer. Deep understanding of how to initiate and modulate tumor-specific immunity would be critical for the development of novel cancer immunotherapies.

In this issue, there are 3 review articles that cover key immunological tools: bispecific antibodies, natural killer (NK) cells, and neoantigens. S. Chen et al. reviewed the current understanding of bispecific antibodies and its chronological development to produce these antibodies, mechanism of action, application in cancer medicine, and the economic impact of these drugs. S. Matosevic reviewed the emergence of NK cells as an adoptive immune-cellular therapy and the technical background behind its engineering from viral and nonviral vectors. R.-Y. Pan et al. reviewed the quest to identify tumor neoantigens and how to leverage these findings in immunotherapy-based platforms in preclinical and clinical models.

In order to complement the aforementioned articles, this issue also included 6 research articles that span different aspects of cancer immunology and therapeutics. I. Poláková et al. demonstrated the utility of deep immune-profiling of lymphoid and myeloid components in the blood and tumor tissue of head and neck cancer patients. Y. Wu et al. identified HLA-A2-based epitopes that can elicit cytotoxic T lymphocyte-based responses. Two monoclonal antibodies targeting EGFR in animal studies were also included here. W. Qiu et al. discussed a novel monoclonal antibody targeting EGFR with better safety and efficacy profiles than cetuximab when combined with irinotecan in animal models. Y. Yang et al. demonstrated the efficacy of fusing *Pseudomonas*-based immunotoxin with a novel EGFR-based antibody in a murine model of esophageal cancer. Q. Zhang et al. demonstrated how combining the multikinase inhibitor, regorafenib, with CAR-NK cells could elicit responses in colorectal cancer cell lines.

Collectively, these articles integrate different aspects of immune-based therapies in the current management of cancer. Our current understanding of immune therapies and antitumoral immunity still requires significant refining. More studies are needed to identify the biomarkers of response to immunotherapies and CAR-based treatments so that nonresponders could be spared the risks of adverse events. Are there other checkpoint blockers that one could leverage in cancer treatment? Which patients would benefit from bispecific antibodies compared to CAR-based treatments? Is there a role for combination immune-based therapies with targeted therapy or chemotherapy? These are questions that are relevant in the decision process for cancer patients and require more in-depth and extensive research to address. Nevertheless, the current evidence strongly suggests that immune-based therapies work but understanding the context of its activity is vital for its success.

